# Imidacloprid modifies the mitotic kinetics and causes both aneugenic and clastogenic effects in the macrophyte *Bidens laevis* L.

**DOI:** 10.1016/j.heliyon.2019.e02118

**Published:** 2019-07-24

**Authors:** Germán Lukaszewicz, Fernando G. Iturburu, Daniela S. Garanzini, Mirta L. Menone, Stephan Pflugmacher

**Affiliations:** a Laboratorio de Ecotoxicología, Instituto de Investigaciones Marinas y Costeras (IIMYC), Consejo Nacional de Investigaciones Científicas y Técnicas (CONICET), Universidad Nacional de Mar del Plata, Funes 3350, 7600 Mar del Plata, Buenos Aires, Argentina; b University of Helsinki, Faculty of Biological and Environmental Sciences, Ecosystems and Environment Research Programme, Aquatic Ecotoxicology in an Urban Environment, Niemenkatu 73, 15140 Lahti, Finland; c Joint Laboratory of Applied Ecotoxicology, Environmental Safety Group, Korea Institute of Science and Technology Europe (KIST Europe) Forschungsgesellschaft mbH, Universität des Saarlandes Campus E7 1, Saarbrücken, 66123, Germany; d Helsinki Institute of Sustainibility, Fabianinkatu 33, 00014 Helsinki, Finland

**Keywords:** Ecology, Environmental science, Ecological health, Environmental impact assessment, Water pollution, Environmental toxicology, Plant Genetics, Photosynthesis, Mutation, DNA damage, Neonicotinoids, Cell cycle, Aquatic macrophyte

## Abstract

Imidacloprid (IMI) is a neonicotinoid insecticide widely used in agricultural activities all around the world. This compound is transported from croplands to surrounding freshwater ecosystems, producing adverse effects on non-target organisms. Because of the relevance of aquatic macrophytes in the above-mentioned environments and the lack of studies of potential effects of IMI on them, this work aimed to assess the mitotic process and potential genotoxicity in the aquatic macrophyte *Bidens laevis* L. Although the analysis of the Mitotic Index (MI) showed that IMI was not cytotoxic, the Cell Proliferation Kinetics (CPK) frequencies evidenced modifications in the kinetics of the mitotic process. Indeed, the anaphases ratio decreased at 10 and 100 μg/L IMI, while at 1000 μg/L an increase of prophases ratio and a decrease of metaphases ratio were observed. Regarding genotoxicity, IMI produced an increase of the abnormal metaphases frequency from 10 μg/L to 1000 μg/L as well as an increase in clastogenic anaphases-telophases frequency at 100 and 1000 μg/L. In addition, aneugenic anaphases-telophases and C-mitosis frequencies also increased at 1000 μg/L, confirming the effects on the mitotic spindle. Considering the genotoxic effects on *B. laevis* through two different mechanisms (aneugenic and clastogenic) and the wide spread use of IMI in agriculture, these mechanisms of toxicity on macrophytes should be considered among other recognized effects of this insecticide on aquatic biota.

## 1 Introduction

Among current use pesticides, neonicotinoids chemical group sales grew the most in recent years (Elbert et al., 2008). Their chemical properties (mainly their hydrophilicity) allow systemic protection of crops, leading to new modes of application, i.e. seed coating (Bonmatin et al., 2015). Nevertheless, this hydrophilicity let them reach water bodies by runoff or lixiviation events (Miles et al., 2017). Even falling leaves from trees have been reported to contribute to the entry of neonicotinoids into water ecosystems (Englert et al., 2018). Mainly, imidacloprid (IMI) was the first launched neonicotinoid and nowadays it is the best-selling insecticide in the world. The presence of this compound in water has been reported in different water bodies around the world: from wetlands in Canada (Main et al., 2014) to different basins in Argentina Pampas (De Gerónimo et al., 2014) in America, and there are also reports from Australia, Europe and Asia, with maximum found concentrations of 4.56 (Sanchez-Bayo and Hyne, 2014), 15 (Kreuger et al., 2010) and 0.19 μg/L IMI (Lamers et al., 2011), respectively. Even higher concentrations were detected in aquatic environments reaching 320 μg/L IMI in Netherlands, as reported by van Dijk et al. (2013). For a complete review of neonicotinoids in surface water please see Morrissey et al. (2015).

Even if the primary concern in risk assessment is toxicity of IMI and other neonicotinoids on pollinators (van Lexmond et al., 2015), there is abundant evidence of its lethal and some sublethal effects on aquatic organisms (for a complete review see Anderson et al., 2015). Imidacloprid is an agonist of postsynaptic acetylcholine nicotinic receptors (nAchR), being invertebrates more sensitive to its toxic effects than vertebrates because invertebrates' nAchR have a lower higher affinity for IMI than their vertebrate homologues (Tomizawa and Casida, 2005). However, IMI could also affect aquatic vertebrates directly (mainly sublethal effects to environmentally relevant concentrations) or indirectly (i.e. lack of preys, Gibbons et al., 2015). In addition to its own effects on biota, IMI environmental metabolites could be more toxic to vertebrates than the parental compound (for example desnitro- IMI, Tomizawa and Casida, 2005). All these effects (both on invertebrates and vertebrates) have led to negative consequences to aquatic communities as well as to the ecosystem level (Sanchez-Bayo et al., 2016). Although a lack of information about toxicity mechanisms of IMI on non-target organisms is recognised, none of the efforts is focused on photosynthetic components of freshwater ecosystems. Aquatic macrophytes represent the main photosynthetic organisms in freshwater aquatic ecosystems for being, not only primary producers but also for providing other critical ecological services (i.e. nutrients cycling, habitat provision, Gopal, 2016). Because these organisms are exposed to pollutants from both sediment and water column, they can indicate possible contamination in these matrices and constitute important biomonitor species in ecotoxicological studies (Monferrán et al., 2009).

DNA damage is an early biological effect which could disturb biological structures and functions and lead to a genotoxic syndrome related to carcinogenic problems (Anderson et al., 1994). A recent review has shown the broad spectrum of species which suffer carcinogenic processes due to several reasons, including DNA damage produced by chemical pollution (Pesavento et al., 2018). Moreover, unrepaired/misrepaired DNA damage in germ cells of natural biota could have effects on fitness and reproductive success, and it would ultimately lead to long-term deterioration of the ecosystem quality (Jha, 2008).

Most plant genotoxicity bioassays are developed with terrestrial model species as *Allium cepa* or *Tradescantia palida*. There are few approaches to use wildlife species for these assessments, as Gadeva and Dimitrov (2008) study using *Crepis capillaris* (Fam. Asteraceae) to test possible genotoxic effects of pesticides on root-meristem cells. However, these attempts do not include the use of wetland macrophytes, which could offer a more realistic scenario for aquatic environmental genotoxicity studies. A standardised plant bioassay was initially developed for *A. cepa* to assess potential genotoxic effects of chemical compounds or complex solutions, evaluating cytogenetic biomarkers such as chromosome aberrations in anaphase–telophase (CAAT) and abnormal metaphases quantification (Rank, 2003). These biomarkers allow evaluating both spindle disturbance (aneunogenesis) and DNA strand break (clastogenicity) in growing root tips. This bioassay was adapted to use the wetland macrophyte *Bidens laevis* to assess freshwater pollution, both in laboratory bioassay and for *in situ* biomonitoring (Pérez et al., 2008). *B. laevis* can be found in shallow freshwaters, widely spread from the southern USA to South America. In Argentina, it inhabits marsh and stream edges in several regions including the Pampas, with extensive agricultural activities. Its genetic characteristics (chromosomes size and number) turned it into a suitable species for genotoxicity assessing (Menone et al., 2015).

Mainly, reports of genotoxic effects of IMI on photosynthetic organisms are scarce, and they focus on terrestrial plants, as *A. cepa* or *Tradescantia pallida* (Ansoar-Rodriguez et al., 2015). Regarding aquatic organisms, IMI genotoxicity was only reported on animals, such as frog tadpoles (Pérez- Iglesias et al., 2014) and fishes (Iturburu et al., 2017, 2018), but there are no available reports of this effect on aquatic macrophytes. Given the importance of aquatic macrophytes in the aquatic ecosystems, this work aimed to assess the potential adverse effects of IMI on the mitotic process and DNA integrity (evaluated through CAAT and abnormal metaphases frequencies quantification) in the aquatic macrophyte *B. laevis*.

## 2 Material and methods

### 2.1 Chemicals

The pesticide studied was IMIDA NOVA 35 (Imidacloprid 350 g/L) as a commercial formulation. Positive control, Methyl Methanesulfonate (MMS, Sigma Aldrich®) was used as an inductor of chromosomal aberrations and dimethyl formamide (DMF, Dorwil®) was used for pigment extraction. Other reagents were of the highest purity available (Hoagland's salts, ethanol and acetic acid from Biopack®, orcein from Sigma Aldrich® and HCl from Dorwil®).

### 2.2 Biological material

Seeds of *B. laevis* were collected in Tajamar stream (37° 52′ 57″S, 57° 54′ 46″W), Argentina in May 2015. They were sterilized with a solution of chlorine (10%) and distilled water for 10 minutes and washed thoroughly with distilled water. The seeds were scarified under magnifying glass and placed in Petri dishes with humid filter paper for germination. After 3–4 days the seedlings were moved to soil-containing pots and placed in a growth chamber at 24 ± 2 °C and 16/8 h light/darkness photoperiod for 45 days until exposure.

### 2.3 Exposure conditions

A single experiment was carried out, in which six solutions were tested, four of which had a final concentration of IMI of 1, 10, 100 and 1000 μg/L, one negative control (C (-)) consisting of Hoagland solution (Hoagland and Arnon, 1950) and one positive control (C (+)) consisting of 10 mg/L MMS. All IMI and MMS solutions were dissolved in Hoagland solution. For each treatment a set of six plants was tested (n = 6). All plants were exposed individually in glass recipients with 300 mL of the corresponding solution, with the roots submerged and the stem and leaves out of the exposure solution. Exposure time was 24 h, followed by another 24 h in Hoagland solution to allow a complete cell cycle (Pérez et al., 2008). Exposures were carried out in static conditions, since IMI showed stability, at least for 48 h, in water in previous bioassays performed in our laboratory with the same concentrations (Iturburu et al., 2017).

### 2.4 Evaluation of chlorophylls concentration

In order to assess the physiological status of the plants, the chlorophyll content in leaves was evaluated according to Inskeep and Bloom (1985). Leaf samples (2 leaves per plant) of 0.2 g each were put into vials containing 2 ml of N, N-dimethylformamide (DMF), and kept in darkness at 4 °C for 72 h. The absorbance values of the supernatant were recorded at 647 and 664 nm using a spectrophotometer Shimadzu UV-210 A. The equations designed by Wellburn (1994) were applied for calculating the content of chlorophyll *a*, *b*, and total, as well as the *a*/*b* ratio.

### 2.5 Evaluation of mitotic kinetics and genotoxicity

Roots of 1 cm length were collected and immersed in fixation solution (ethanol: glacial acetic acid, 3:1) for 24 h to stop the cell cycle, then moved to 70% ethanol solution and kept at 5 °C until microscopic analysis. Two roots per sample were hydrolyzed in HCl 1N during 10 min, and the root tips were stained with acetic orcein 2% during 10 min in order to prepare each slide. For every sample/slide a total of 1000 cells were analyzed to determine the mitotic index (MI) as the percentage of dividing cells. Cell Proliferation Kinetics (CPK) frequencies were calculated as the number of cells in each division phase over total mitotic cells. Chromosomal Aberrations in Anaphase-Telophase (CAAT) frequency was determined as the ratio of aberrant cells over 200 anaphase/telophase cells per plant. Aberrations were grouped according to their origin in clastogenic (causing chromosomal breakage) or aneunogenic (disturbing spindle function and thus causing asynchronic chromosomal migration). Regarding aneunogenic aberrations, laggard and vagrant chromosomes and tripolar figures have been scored. Within clastogenic aberrations, bridges, fragments, double bridges, double fragments and rings were considered. Abnormal metaphases (AM) frequency was scored as non-congregated metaphase chromosomes over 100 total metaphases per plant. C-mitosis, described by Grant (1978) as an inactivation of the spindle followed by a random scattering of the chromosomes over the cell, were scored and quantified by calculating the frequency over 100 metaphases per plant. MI, CPK frequencies, CAAT, AM and C-mitosis were scored in a single slide per plant unless the minimum number of cells for each counting could not be reached, in which case supplemental slides were prepared with the fixed roots, as previously explained.

### 2.6 Statistical analysis

Normality and homogeneity of variances were verified with D'agostino- Pearson and Barlett's test, respectively. Chlorophylls content, MI and CPK, which showed a normal distribution of data, were compared with ANOVA test and a *post hoc* Dunnett's test to compare each treatment with its controls. The remaining data (CAAT, AM and C-mitosis) did not pass normality test and were square root transformed and analyzed as mentioned before. Statistical analysis were performed with GraphPad Prism v6.01 (GraphPad Software, Inc.) using a confidence level of 95%.

## 3 Results

Leaves *of B. laevis* showed no changes on chlorophyll *a* and *a/b* ratio with respect to control (p > 0.05), although an increase of chlorophyll *b* and total chlorophyll content were detected at 1000 and 1 and 1000 μg/L IMI, respectively (p < 0.05, Table 1).

**Table 1:** Chlorophylls content in leaves of *Bidens laevis* exposed to imidacloprid.

[IMI] (μg/L)	[Chl *a*]	[Chl *b*]	*a/b* ratio	[Total Chl]
C (-)	617 ± 176	235 ± 76	2.7 ± 0.4	853 ± 243
1	798 ± 124	375 ± 47	2.1 ± 0.3	1173 ± 151*
10	682 ± 101	278 ± 90	2.6 ± 0.8	959 ± 166
100	702 ± 129	289 ± 105	2.6 ± 0.6	991 ± 213
1000	723 ± 72	433 ± 147*	1.9 ± 0.7	1156 ± 171*

Values are expressed as mg/g fresh weight (mean ± SD). C (-): negative control. *: significantly different from C (-) (p < 0.05, ANOVA and Dunnett's *post hoc* tests).

There were no evident effects on the MI in roots at any IMI concentrations tested (p > 0.05) (Table 2). On the other hand, CPK (evaluated as ratio of prophases, metaphases, anaphases and telophases) showed an increase in the ratio of prophase and a decrease in metaphase ratio at 1000 μg/L IMI (p < 0.05, Table 2). In addition, in plants exposed to 10 and 100 μg/L IMI a decrease in the anaphase ratio was detected (p < 0.05, Table 2).

**Table 2:** Mitotic Index and Cell Proliferation Kinetics (ratio of phases) in *Bidens laevis* exposed to imidacloprid.

[IMI] (μg/L)	Mitotic Index	Prophases	Metaphases	Anaphases	Telophases
C (-)	9.5 ± 1.3	50.4 ± 4.9	20.6 ± 6.8	13.1 ± 3.0	15.9 ± 3.7
1	8.2 ± 0.9	51.7 ± 4.7	16.2 ± 1.8	12.4 ± 2.3	18.6 ± 1.5
10	8.9 ± 1.3	53.3 ± 2.5	17.3 ± 4.5	8.3 ± 1.9*	19.7 ± 3.0
100	8.7 ± 0.7	56.8 ± 2.1	15.2 ± 3.7	8.1 ± 1.8*	20.8 ± 2.5
1000	10.1 ± 0.9	59.3 ± 2.6*	11.3 ± 1.1*	10.7 ± 2.5	19.9 ± 3.7

Values are expressed as % (mean ± SD) C (-): negative control. *: significantly different from C (-) (p < 0.05, ANOVA and Dunnett's post hoc tests).

**Fig. 1:**
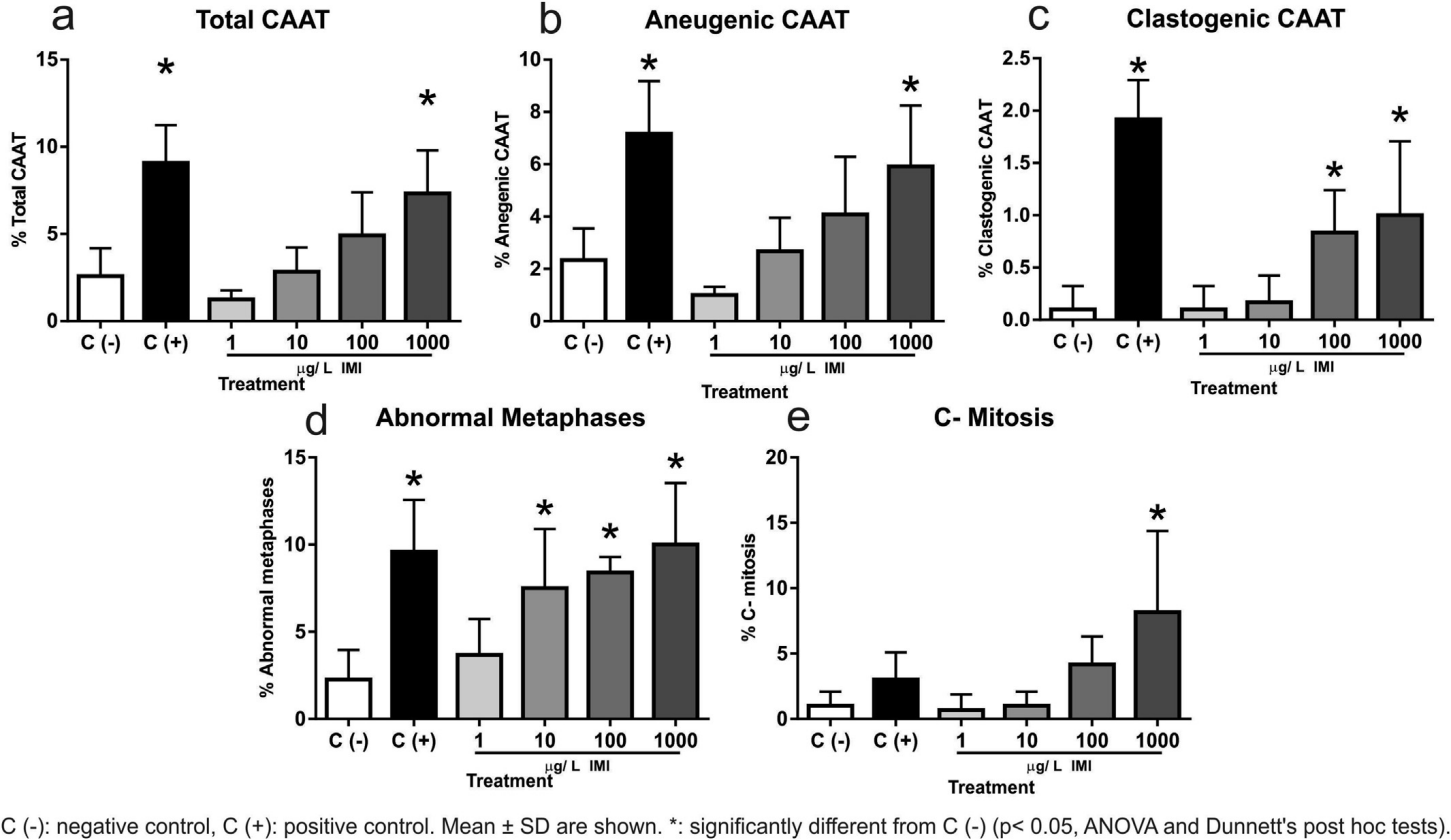
Biomarkers of genotoxicity in the aquatic macrophyte *Bidens laevis* exposed to imidacloprid.

**Fig. 2:**
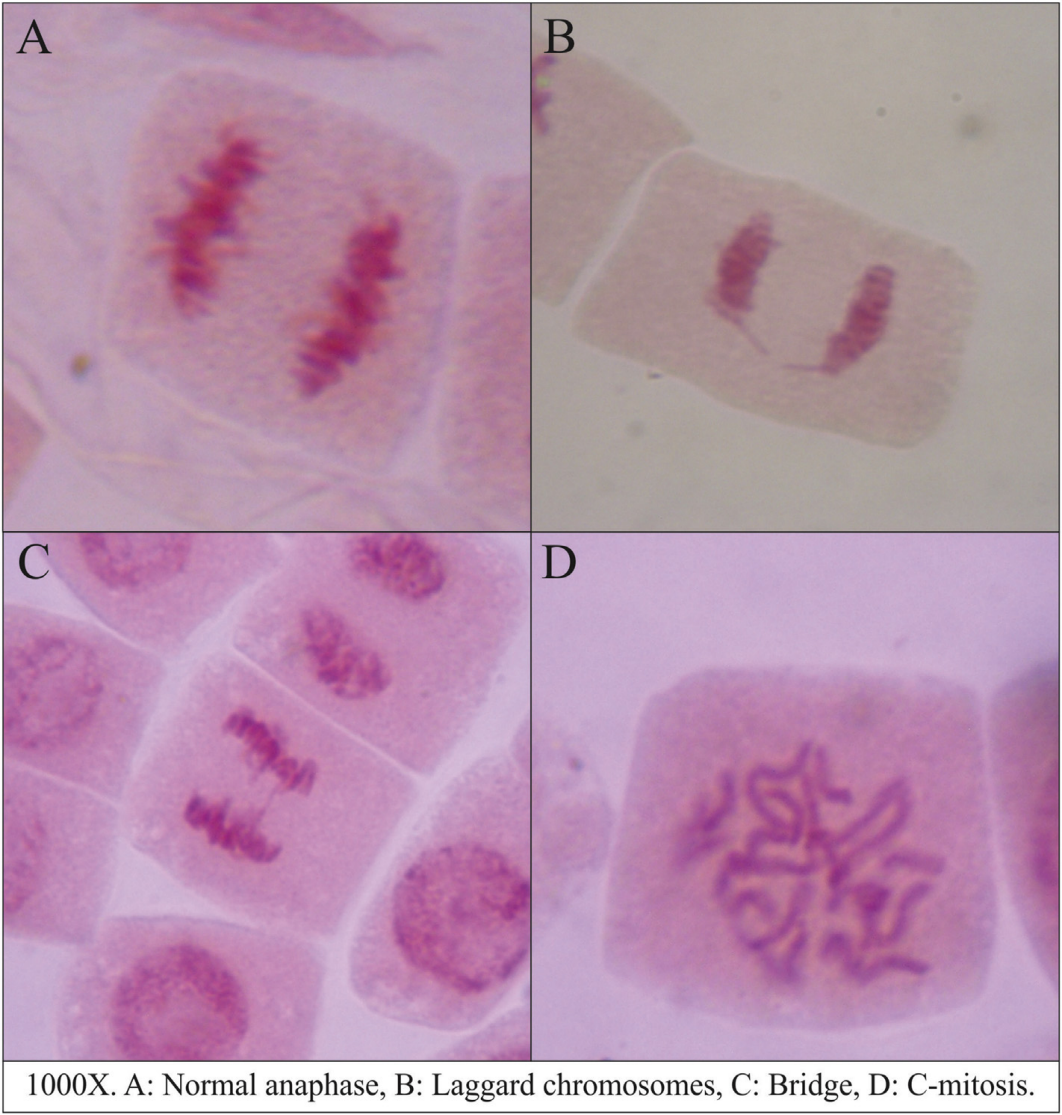
Microphotographs of chromosomal abnormalities in root cells of *Bidens laevis* exposed to imidacloprid.

A 24 h exposure to the recognized genotoxic compound MMS produced a significant increase of aneugenic, clastogenic and total CAAT figures (3, 19 and 3.5-fold, respectively; p < 0.05, Fig. 1) as well as an increase in the frequency of abnormal metaphases (4.3- fold, p < 0.05, Fig. 1d). On the other hand MMS did not increase the frequency of C-mitosis (p > 0.05, Fig. 1e). The most frequent aberrations observed among all treatments in anaphase-telophase were laggards and vagrants chromosomes, as well as chromosome bridges (Fig. 2). Plants exposed to 1000 μg/L IMI showed a higher number of aneugenic figures than negative control (p < 0.05, Fig. 1b). In plants exposed to IMI, an increase in clastogenic figures was observed at 100 and 1000 μg/L IMI (p < 0.05, Fig. 1c). Considering the frequency of total CAAT, a significant increase was observed in plants exposed to 1000 μg/L IMI (p < 0.05, Fig. 1a). Finally, abnormal metaphases frequency increased in plants exposed to 10, 100 and 1000 μg/L IMI (p < 0.05, Fig. 1d), while C-mitosis increased at 1000 μg/L IMI (p < 0.05, Fig. 1e).

## 4 Discussion

Total chlorophylls content in control plants showed values similar or even over the previously detected ones in *B. laevis* (Moreyra et al., 2019), denoting an optimal physiological status. Regarding disturbance of the photosynthesis, to the best of our knowledge, there are scarce reports of neonicotinoids effects on this process in wetland macrophytes, as for example a decrease in photosynthetic rate and chlorophyll content in rice (*Oryza sativa* L.) treated with 30 and 60 mg/L imidacloprid (Cheng et al., 2012). On the other hand, effects in terrestrial plants are controversial; while Gonias et al. (2008) found higher levels of photosynthesis in imidacloprid-treated cotton plants (*Gossypium hirsutum* L.), Preetha and Stanley (2012) observed no variations in chlorophyll content in the same species. While our results are clear, a more comprehensive battery of biomarkers is necessary to understand if the whole photosynthetic process is affected by IMI.

The MI, evaluated as a biomarker of cell proliferation in root tips ranged between 8.3- 10.3 % (median values), regardless of the exposure treatment. These values were high enough to score the required number of cells for the evaluation of genotoxicity. Moreover, they were over the range presented previously in controls under similar exposure conditions (7.5 % Pérez et al., 2011; and 6.5 % Pérez et al., 2014). The lack of IMI effect on MI is in agreement with previous studies in *A. cepa*, where no adverse effects on MI were found (Ansoar-Rodriguez et al., 2015; Bianchi et al., 2016). Although the analysis of the MI showed that IMI was not cytotoxic, the CPK frequencies evidenced modifications in the kinetics of the mitotic process. Indeed, the anaphases ratio decreased at 10 and 100 μg/L IMI, while at 1000 μg/L an increase of prophases ratio and a decrease of metaphases ratio were observed. These effects on the ratio of mitotic phases indicates a miscoordination on the mitosis process. Although we do not know if IMI can affect the transition from prophase to metaphase, affecting the spindle assembly checkpoint (SAC) or causing a partial or entire inactivation of the spindle microtubules, it seems to be clear that from 10 μg/L IMI the increase in abnormal metaphases observed can therefore, explain the reduction of the following stage, the anaphases. Recently, it has been demonstrated that *Arabidopsis* delays mitosis in a SAC-dependent manner if the spindle is perturbed, particularly under microtubule-destabilising conditions (Komaki and Schnittger, 2017), a fact that could perhaps take place in *B. laevis* exposed to IMI. The mechanisms involved deserves an in-depth study that goes beyond the objectives of the present work, but the effects observed becomes particularly relevant, since they took place at environmentally relevant concentrations (e.g. 15 μg/L in freshwater ecosystems from Sweden, Kreuger et al., 2010). The observed increase of C-mitosis figures could be *a priori* related to a metaphase arrest on root cells, effect typically observed in cells treated with colchicine (Doležel et al., 2014). However metaphase arrest is usually related to an increase in the number of prophases and metaphases and a consequent decrease of anaphases and telophases, which was not consistently evidenced when *B. laevis* cells were exposed to IMI.

Regarding genotoxicity, IMI produced an increase of the abnormal metaphases frequency from 10 μg/L to 1000 μg/L as well as an increase in clastogenic anaphases-telophases frequency at 100 and 1000 μg/L. In addition, aneunogenic anaphases-telophases and C-mitosis frequencies also increased at 1000 μg/L, confirming the effects on the mitotic spindle. Regarding the aneunogenic effects reported for IMI, *in vitro* studies have demonstrated that this neonicotinoid insecticide could lead to chromosome missagregation and aneuploidy induction in human peripheral blood lymphocytes *in vitro* (Mužinic et al., 2018). In plants, the aneuploid daughter cells originated are typically removed by apoptosis, but effects on spindle could also lead to a polyploidization process, being this a dominant driving force in plant evolution (Komaki and Schnittger, 2017). In the case of *B. laevis,* these possible consequences should be investigated in future studies to confirm these hypotheses.

On the other hand, a possible cause of CPK disarrangement could be related to the DNA clastogenicity produced by IMI. Plants DNA stress checkpoint regulators can sense DNA damage to maintain the genome integrity and to modulate the cell cycle (Hu et al., 2016). DNA structure damage produced by 1000 μg/L was previously observed in other aquatic organisms. Iturburu et al. (2018) found that IMI produced an increase of DNA damage on red cells of the cichlid *Australoheros facetus*, caused by DNA oxidation at concentrations from 1 to 1000 μg/L.

Toxicity of IMI on aquatic photosynthetic organisms has been scarcely studied. Specifically, the available information is limited to growth inhibition of the macrophyte *Lemna gibba* (EC_50_ = 740 mg/L; Daam et al., 2013) and unicellular algae (LC_50_ = 20 mg/L for *Chlamydomonas mexicana* and NOEC = 10 mg/L IMI for *Scenedesmus subspicatus*; Kumar et al., 2015; IUPAC PPDB, 2018). Particularly, genotoxicity in photosynthetic organisms has been reported in terrestrial model species. Bianchi et al. (2016) found an increase in chromosomal aberrations in roots of *A. cepa* after 24 h of exposure to 36 mg/L IMI, a concentration certainly over the range of the realistic ones. Similar results have been reported by Ansoar-Rodriguez et al. (2015) when they exposed *A. cepa* to IMI concentrations typically used in sugarcane crops.

DNA damage could lead to genomic instability or genotoxic stress, triggering diseases, senescence, cellular ageing or changes in gene expression (Patnaik et al., 2011). An increase in the genomic instability has been suggested as a cause of the decrease of population fitness, both in animals and plants (Jha, 2008). Since genotoxic agents may exert damage beyond that of individuals and may be detected through several generations, genotoxicity biomarkers should be considered to evaluate possible toxic effects in aquatic organisms (Frenzilli et al., 2009). Imidacloprid genotoxicity on aquatic organisms becomes relevant, both for its reported worldwide presence in water bodies and also for its genotoxic consequences in organisms.

## 5 Conclusion

The results shown in the present study demonstrate that IMI is able to produce modifications in the kinetics of the mitotic process in root tip cells, and also to cause genotoxic effects in the macrophyte *B. laevis* through aneugenic and clastogenic mechanisms. These results should raise our concern about the negative effects of agricultural pesticides on non-target organisms inhabiting wetlands.

## Declarations

### Author contribution statement

Germán Lukaszewicz: Conceived and designed the experiments; Performed the experiments; Analyzed and interpreted the data; Wrote the paper.

Fernando G. Iturburu, Daniela S. Garanzini: Performed the experiments; Analyzed and interpreted the data; Wrote the paper.

Mirta L. Menone: Conceived and designed the experiments; Analyzed and interpreted the data; Contributed reagents, materials, analysis tools or data; Wrote the paper.

Stephan Pflugmacher: Conceived and designed the experiments; Wrote the paper.

### Funding statement

This work was supported by FONCYT (PICT 2013- 1348), UNMDP (EXA 795/16) and PROALAR Program Mincyt- DAAD (Project DA/13/04).

### Competing interest statement

The authors declare no conflict of interest.

### Additional information

No additional information is available for this paper.
